# Isoxazolidine Conjugates of N3-Substituted 6-Bromoquinazolinones—Synthesis, Anti-Varizella-Zoster Virus, and Anti-Cytomegalovirus Activity

**DOI:** 10.3390/molecules23081889

**Published:** 2018-07-28

**Authors:** Magdalena Grabkowska-Drużyc, Graciela Andrei, Dominique Schols, Robert Snoeck, Dorota G. Piotrowska

**Affiliations:** 1 Bioorganic Chemistry Laboratory, Faculty of Pharmacy, Medical University of Lodz, Muszyńskiego 1, 90-151 Lodz, Poland; magdalena.grabkowska-druzyc@umed.lodz.pl; 2 Rega Institute for Medical Research, KU Leuven, Herestraat 49, B-3000 Leuven, Belgium; Graciela.Andrei@rega.kleuven.be (G.A.); Dominique.Schols@rega.kleuven.be (D.S.); Robert.Snoeck@rega.kleuven.be (R.S.)

**Keywords:** phosphonate, isoxazolidine, quinazolinones, antiviral, cytostatic

## Abstract

1,3-Dipolar cycloaddition of *N*-methyl *C*-(diethoxyphosphoryl) nitrone to N3-substituted 6-bromo-2-vinyl-3*H*-quinazolin-4-ones gave (3-diethoxyphosphoryl) isoxazolidines substituted at C5 with quinazolinones modified at N3. All isoxazolidine cycloadducts were screened for antiviral activity against a broad spectrum of DNA and RNA viruses. Several isoxazolidines inhibited the replication of both thymidine kinase wild-type and deficient (TK^+^ and TK^−^) varicella-zoster virus strains at EC_50_ in the 5.4–13.6 μΜ range, as well as human cytomegalovirus (EC_50_ = 8.9–12.5 μΜ). Isoxazolidines *trans*-**11b**, *trans*-**11c**, *trans*-**11e**, *trans*-**11f**/*cis*-**11f**, *trans*-**11g**, *trans*-**11h**, and *trans*-**11i**/*cis*-**11i** exhibited moderate cytostatic activity towards the human lymphocyte cell line CEM (IC_50_ = 9.6–17 μM).

## 1. Introduction

Herpesviruses are enveloped, icosahedral viruses containing double-stranded DNA. What fundamentally distinguishes herpesviruses from others is the ability to establish latent infection and reactivating when the host immune system is impaired. Human cytomegalovirus (HCMV) and varicella zoster virus (VZV) belong to the family of herpesviruses which cause infections usually acquired in childhood [1,2,3]. For the treatment of viral infections caused by herpesviruses, including VZV and HCMV, several drugs, such as acyclovir (e.g., *Zovirax*), valacyclovir (e.g., *Valtrex*), famciclovir (e.g., *Famvir*), brivudin (e.g., *Zostex*, *Helpin*), and ganciclovir (e.g., *Cymevene*), have been used [3,4,5].

Despite the wide range of available drugs, effective treatment of viral infections is still limited. This is mainly due to the specificity of viral infections and the ability of viruses to mutate, which is associated with the acquisition of drug resistance. Therefore, new compounds with potential biological activity are being sought all the time. Quinazolinone and its derivatives have been extensively studied because of their wide range of biological activities, including antiviral [6], anticancer [7,8], antifungal [9], antibacterial [10,11], antitubercular [12,13], anti-inflammatory [11,14], anticonvulsant [15], hypolipidemic [16], analgesic [17], or immunotropic properties [18].

**Figure 1:**
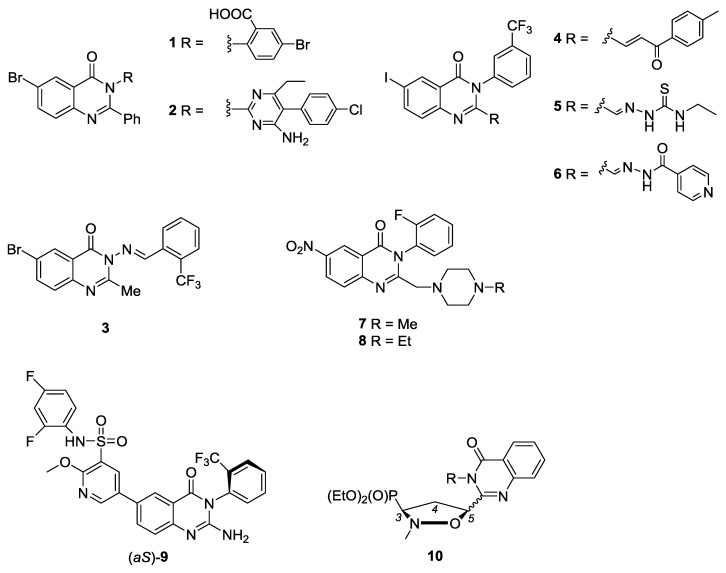
Examples of C6-substituted quinazolinone derivatives with antiviral activity.

The antiviral activity of compounds containing the C6-substituted quinazolin-4-one framework has been discovered in recent years (Figure 1). For example, 5-bromo-2-(6-bromo-4-oxo-2-phenyl-4*H*-quinazolin-3-yl)-benzoic acid **1** was reported to possess distinct antiviral activity against both herpes simplex viruses 1 and 2 (HSV-1 and HSV-2) and vaccinia virus (VACV) (EC_50_ = 12 μg/mL) [19], whereas compound **2** exhibited activity against vaccinia virus in E_6_SM cell cultures (MIC of 1.92 μg/mL) [20]. Another analogue, i.e., 2-methyl-3-(substituted-benzalamino)-4(3*H*)-quinazolinone **3**, was found to exhibit antiviral activity against tobacco mosaic virus (TMV) in vivo. Compound **3** showed curative effects of 54%, which was slightly higher than that of a reference drug, Ningnanmycin [21]. Very recently quinazolin-4(3*H*)-ones **4**–**6** were synthesized and evaluated for inhibitory action on the replication of influenza A virus (H_5_N_1_), as well as to test toxicity on in vitro cell lines. In general, quinazolin-4(3*H*)-ones containing a chalcone skeleton **4**, thiosemicarbazone **5**, and hydrazide **6** showed moderate antiviral activity against H_5_N_1_ (inhibition rate: 38%, 47%, 25%, respectively, for **4**, **5**, **6**) compared to a reference drug, Zanamivir [22]. In addition, compounds **7** and **8** possessing a nitro group at C6 were reported as potent inhibitors of Venezuelan Equine Encephalitis Virus (VEEV) (EC_50_ = 0.8 μM, CC_50_ = 50 μM) [23]. Quinazolinone (*aS*)-**9** displayed high selectivity for PI4KIIIα and appeared to be potent inhibitors of hepatitis C virus (HCV) replication in vitro. Moreover, compound (*aS*)-**9** exhibited higher potency for PI4KIIIα than its atropisomer *aR* (pIC_50_ of 8.3 vs. 6.9) and an improved selectivity range (2.7–3.2 vs. 1.4–2.1) against the other lipid kinases [24].

**Scheme 1:**
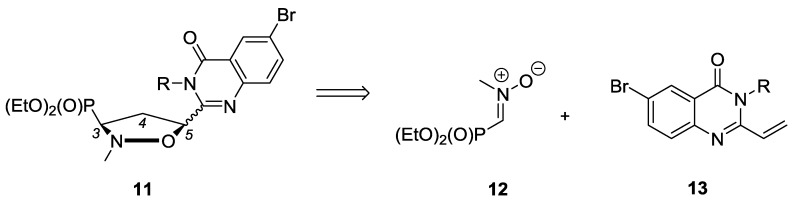
Retrosynthesis of isoxazolidine conjugates of 6-bromoquinazolinones.

Recently, we have succeeded in synthesis of a series of quinazolinones substituted at C3 with a (diethoxyphosphoryl)isoxazolidine moiety **10**. Several of the synthesized analogues exhibited moderate activity against varicella zoster virus (VZV) and human cytomegalovirus (HCMV) [25]. On the basis of these observations, and in continuation of our search for new biologically active compounds [25], a novel series of quinazolinone derivatives of general formula **11** having an isoxazolidine ring at C2, different substituents at N3, and an additional bromine atom at C6 was synthesized. The synthetic strategy for our new isoxazolidine-conjugates of quinazolinones relies on the 1,3-dipolar cycloaddition of *N*-methyl-*C*-(diethoxyphosphoryl)nitrone **12** [26] with selected 6-bromo-2-vinyl-3*H*-quinazolin-4-ones **13** (Scheme 1).

## 2. Results and Discussion

### 2.1. Chemistry

**Scheme 2:**
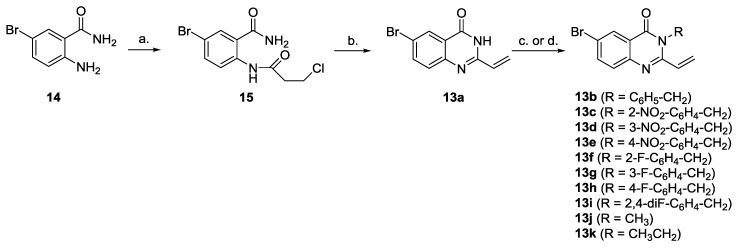
Synthesis of 3-substituted 6-bromo-2-vinylquinazolinones **13a**–**13k**. Reaction and conditions: a. 3-chloropropionyl chloride, 1,4-dioxane, 0 °C; b. 5% NaOH–EtOH (2:1); c. RBr, KOH, 105 °C; d. MeI or EtI, KOH, CH_3_CN, 60 °C.

The respective 3-substituted 6-bromo-2-vinyl-3*H*-quinazolin-4-ones **13a**–**k** were synthesized starting from the commercially available isatoic acid anhydride, which was first reacted with bromine and then transformed into 5-bromoanthranilic acid amide **14** according to the procedure in the literature [27,28]. Reaction 5-bromoanthranilic acid amide **14** with 3-chloropropionyl chloride followed by cyclization and dehydrohalogenation led to the formation of 6-bromo-2-vinyl-3*H*-quinazolin-4-one **13a**, which was then used as a key intermediate in the preparation of N3-substituted derivatives **13b**–**13k** via alkylation with the respective benzyl bromides, as well as with methyl or ethyl iodide [29] (Scheme 2).

**Scheme 3:**
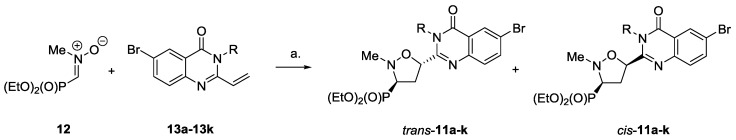
Synthesis of isoxazolidines *trans*-**11a**–**k** and *cis*-**11a**–**k**. Reaction and conditions: a. toluene, 70 °C, 24 h.

The 1,3-dipolar cycloadditions of nitrone **12** with the respective 6-bromo-2-vinylquinazolinones **13a**–**13k** were carried out at 70 °C in toluene and afforded mixtures of diastereoisomeric isoxazolidines *trans*-**11a**–**11k** and *cis*-**11a**–**11k** (Scheme 3, Table 1). Ratios of trans/cis diastereoisomers were determined on the basis of the ^31^P-NMR spectra of crude products and confirmed by the analysis of ^1^H-NMR spectra data. In all cases good trans/cis diastereoselectivities were observed (in the range of 76–88%) and *trans*-isomers predominated. The mixtures of isoxazolidine cycloadducts were separated chromatographically on silica gel. The isolation of pure isomers was successfully accomplished for the major isomers *trans*-**11a** (R = H), *trans*-**11b** (R = C_6_H_5_CH_2_), *trans*-**11c** (R = 2-NO_2_-C_6_H_4_-CH_2_), *trans*-**11d** (R = 3-NO_2_-C_6_H_4_-CH_2_), *trans*-**11e** (R = 4-NO_2_-C_6_H_4_-CH_2_), *trans*-**11g** (R = 3-F-C_6_H_4_-CH_2_), *trans*-**11h** (R = 4-F-C_6_H_4_-CH_2_), *trans*-**11j** (R = CH_3_), and *trans*-**11k** (R = CH_3_CH_2_), as well as for a minor isomer *cis*-**11a** (R = H).

Stereochemistry of the cycloaddition of a nitrone **12** to N3-substituted derivatives of 2-vinyl-3*H*-quinazolin-4-ones has recently been described and relative configurations of *trans*- and *cis*-isoxazolidine cycloadducts were established based on conformational analysis [25]. Since the introduction of the bromine atom at C6 of the quinazolin-4-one framework has no influence on the stereochemical outcome of the reaction of nitrone **12** with N3-susbstituted 2-vinyl-3*H*-quinazolin-4-ones, the trans and cis configurations of the major *trans*-**11a**–**k** and minor *cis*-**11a**–**k** isomers were assigned taking advantage of our previous observations.

### 2.2. Antiviral and Cytostatic Evaluation

#### 2.2.1. Antiviral Activity

Pure isomers *trans*-**11a**, *trans*-**11b**, *trans*-**11c**, *trans*-**11d**, *trans*-**11e**, *trans*-**11g**, *trans*-**11h**, *trans*-**11j**, *trans*-**11k**, and *cis***-11a**, as well as diastereoisomeric mixtures of isoxazolidines *trans*-**11f/***cis*-**11f** (95:5) and *trans*-**11i/***cis*-**11i** (92:8), were tested for inhibitory activity against a wide variety of DNA and RNA viruses. The following cell-based assays were used: (a) human embryonic lung (HEL) cells (herpes simplex virus-1 (KOS strain), herpes simplex virus-2 (G strain), thymidine kinase deficient (acyclovir resistant) herpes simplex virus-1 (TK^−^ KOS ACV^r^), vaccinia virus, adenovirus-2, vesicular stomatitis virus, cytomegalovirus (AD-169 and Davis strains), varicella-zoster virus (TK^+^ VZV and TK^−^ VZV strains)); (b) HeLa cell cultures (vesicular stomatitis virus, Coxsackie virus B4, and respiratory syncytial virus); (c) Vero cell cultures (parainfluenza-3 virus, reovirus-1, Sindbis virus, Coxsackie virus B4, Punta Toro virus, yellow fever virus); (d) CrFK cell cultures (feline corona virus (FIPV) and feline herpes virus (FHV)); and (e) MDCK cell cultures (influenza A virus (H1N1 and H3N2 subtypes) and influenza B virus). Ganciclovir, cidofovir, acyclovir, brivudin, zalcitabine, zanamivir, alovudine, amantadine, rimantadine, ribavirin, dextran sulfate (molecular weight 10000, DS-10000), mycophenolic acid, Hippeastrum hybrid agglutinin (HHA), and Urtica dioica agglutinin (UDA) were used as the reference compounds. The antiviral activity was expressed as the EC_50_: the compound concentration required to reduce virus plaque formation (VZV) by 50% or to reduce virus-induced cytopathogenicity by 50% (other viruses).

Almost all synthesized isoxazolidines inhibited the replication of both TK^+^ and TK^−^ VZV strains at EC_50_’s in the 5.4–13.6 μΜ range (Table 2), at the same time they were inactive toward other tested viruses, except for HCMV. In general, the inhibitory activity of the tested compounds toward TK^−^ VZV 07-1 strain was better than that of the reference drugs acyclovir and brivudin (EC_50_ = 39.2 μΜ and EC_50_ = 31.9 μΜ, respectively), which require activation by the viral enzyme. On the other hand, the activity of these compounds against the TK^+^ VZV OKA strain was five to nine-fold and 360 to 587-fold lower than that of the reference drugs acyclovir and brivudin, respectively. While exhibiting significant activity toward both TK^+^ and TK^−^ VZV stains, compounds *trans*-**11b**, *trans*-**11d**, *trans*-**11g**, and *trans*-**11h** exhibited the lowest cytotoxicity (CC_50_ = 33–42 μΜ). It is worth mentioning that incorporation of the bromine atom at C6 in the quinazolinone skeleton resulted in a significant increase in potency of isoxazolidine-conjugates **11b**–**11i** against VZV (up to five-fold higher) when compared with previously described analogous conjugates **10b**–**10i** having an unsubstituted quinazoline skeleton [25].

The synthesized isoxazolidine phosphonates also showed marked activity against human cytomegalovirus (HCMV) (Table 3) and, among them, *trans*-**11d**, *trans*-**11g**, *trans*-**11h**, and *trans*-**11i**/*cis*-**11i** (92:8) were the most potent ones. Activities of the tested compounds against both AD-169 and Davis strains were comparable (EC_50_ = 8.9–12.5 μΜ) to that of Ganciclovir (EC_50_ = 16.9 and 7.7 μΜ) used as a reference drug, but still lower than that of the other reference drug, Cidofovir (EC_50_ = 1.5–1.7 μΜ).

#### 2.2.2. Cytostatic Activity

The 50% cytostatic inhibitory concentration (IC_50_) causing a 50% decrease in cell proliferation was determined against murine leukemia L1210, human lymphocyte CEM, human cervix carcinoma HeLa, and immortalized human dermal microvacsular endothelial cells (HMEC-1). Almost all compounds **11a**–**11k** showed inhibitory activity against the proliferation of tumour cell lines (Table 4). They appeared to be the most cytostatic toward the human T-lymphocyte (CEM) cell line with an inhibitory effect in the 9.6–33 μM range. Thus, isoxazolidines *trans*-**11b** (IC_50_ = 16 ± 3 μM), *trans*-**11c** (IC_50_ = 14 ± 4 μM), *trans*-**11e** (IC_50_ = 17 ± 12 μM), *trans*-**11f**/*cis*-**11f** (95:5) (IC_50_ = 13 ± 1 μM), *trans*-**11g** (IC_50_ = 10 ± 0 μM), *trans*-**11h** (IC_50_ = 9.6 ± 2.2 μM), and *trans*-**11i**/*cis*-**11i** (92:8) (IC_50_ = 9.8 ± 4.8 μM) exhibited activity toward the CEM line higher than of 5-fluorouracil (IC_50_ = 18 ± 5 μM).

Structure–activity relationship studies indicated that the isoxazolidine derivatives possessing hydrogen (*cis*-**11a**, *trans*-**11a**), methyl (*trans*-**11j**), or ethyl (*trans*-**11k**) at N3 in the quinazolinone ring were not active against tested viruses and they did not inhibit proliferation of the tested cell lines. Compounds substituted at N3 with benzyl moieties were found effective against VZV, HCMV, and were able to inhibit tumour cell line growth. Moreover, isoxazolidine phosphonates having 3-nitrobenzyl (*trans*-**11d**) and 3-fluorobenzyl (*trans*-**11g**) substituents at N3 of the quinazolinone core showed the highest antiviral activity with an EC_50_ values in the range of 5.8–11.6 μM.

On the other hand, compounds possessing 3-fluorobenzyl (*trans*-**11g**), 4-fluorobenzyl (*trans*-**11h**) and 2,4-difluorobenzyl (*trans*-**11i**/*cis*-**11i** (92:8)) substituents at the N3 position showed an inhibitory effect against the proliferation of murine leukemia (L1210) and human T-lymphocyte (CEM) cell lines, whereas isoxazolidines having 2-nitrobenzyl (*trans*-**11c**) and 2,4-difluorobenzyl (*trans*-**11i**/*cis*-**11i** (92:8)) substituents at the same position of a quinazolinone moiety appeared to be the most active toward the human cervix carcinoma (HeLa) cell line, however, their inhibitory concentration was much lower than that of the reference drugs.

## 3. Experimental Section

### 3.1. General

^1^H-NMR spectra were taken in CDCl_3_ on the following spectrometers: Gemini 2000BB (200 MHz Varian, Palo Alto, CA, USA), and Bruker Avance III (600 MHz, Bruker Instrument, Karlsruhe, Germany) with TMS as internal standard. ^13^C-NMR spectra were recorded for CDCl_3_ solution on the Bruker Avance III at 151.0 MHz. ^31^P-NMR spectra were performed in CDCl_3_ solution on the Varian Gemini 2000 BB at 81.0 MHz or on Bruker Avance III at 243.0 MHz. 

IR spectra were measured on an Infinity MI-60 FT-IR spectrometer (ATI Instruments North America—Mattson, Madison, WI, USA). Melting points were determined on a Boetius apparatus (VEB Kombinat NAGEMA, Dresden, DDR—currently Germany) and are uncorrected. Elemental analyses were performed by the Microanalytical Laboratory of this faculty on a Perkin-Elmer PE 2400 CHNS analyzer (Perkin-Elmer Corp., Norwalk, CT, USA). 

The following adsorbents were used: column chromatography, Merck silica gel 60 (70–230 mesh); analytical TLC, Merck (Merck KGaA, Darmstadt, Germany) TLC plastic sheet silica gel 60 F_254_.

*N*-methyl-*C*-(diethoxyphosphoryl)nitrone [26] and 5-bromoanthranilic acid amide [27,28] were obtained according to the procedures in the literature.

### 3.2. Synthesis of 6-Bromo-2-vinyl-3H-quinazolin-4-one ***13a***

To a solution 5-bromoanthranilic amide (2.40 g, 11.2 mmol) in 1,4-dioxane (5 mL) 3-chloropropionyl chloride (0.527 mL, 5.60 mmol) was added over 20 min. at 0 °C under an argon atmosphere. The mixture was stirred at room temperature for 1 h and then diluted with water until a precipitate appeared, which was collected and washed with water (3 × 10 mL). A mixture of 2-(3-chloropropionylamino)benzamide (2.03 g, 6.63 mmol) in 5% sodium hydroxide (16.4 mL) and ethanol (8.2 mL) was stirred under reflux for 5 min., the solution was allowed to cool for 15 min. and then acidified with acetic acid (1.6 mL). The precipitate was collected and washed with water (3 × 10 mL). The crude 6-bromo-2-vinylquinazolin-4(3*H)*-one was purified by crystallization from methanol. 

Off-white amorphous solid, m.p. = 223–225 °C (re-crystallized from methanol). IR (KBr, cm^−1^) ν_max_: 3165, 3088, 3019, 2943, 2738, 1673, 1638, 1591, 1462, 1337, 1292, 1245, 1207, 1114, 1065, 977, 829, 793, 687, 633. ^1^H-NMR (200 MHz, DMSO): δ = 12.51 (s, 1H, NH), 8.17 (d, ^4^*J* = 2.4 Hz, 1H, *H*C5), 7.90 (dd, ^3^*J* = 8.7 Hz, ^4^*J* = 2.4 Hz, 1H, *H*C7), 7.81 (d, ^3^*J* = 8.7 Hz, 1H, *H*C8), 6.63 (dd, ^3^*J* = 17.5 Hz, ^2^*J* = 3.7 Hz, 1H, CH=C*H*_2_), 6.53 (dd, ^3^*J* = 17.5 Hz, ^3^*J* = 8.1 Hz, 1H, C*H*=CH_2_), 5.86 (dd, ^3^*J* = 8.1 Hz, ^2^*J* = 3.7 Hz, 1H, CH=C*H*_2_). ^13^C-NMR (151 MHz, DMSO): δ = 161.11 (C=O), 152.00, 148.23, 137.80, 131.00, 130.18, 128.45, 126.31, 123.40, 119.37. Anal. Calcd. for C_10_H_7_BrN_2_O: C, 47.84; H, 2.81; N, 11.16. Found: C, 47.88; H, 2.49; N, 11.01.

### 3.3. General Procedure for the Synthesis of N3-Benzylated 6-bromo-2-vinyl-3H-quinazolin-4-ones ***13b***–***13i***

To a solution of 6-bromo-2-vinyl-3*H*-quinazolin-4-one (0.251 g, 1.00 mmol) in acetonitrile (15 mL) potassium hydroxide (0.168 g, 3.00 mmol) was added. After 15 min. the respective benzyl bromide (1.10 mmol) was added and the reaction mixture was stirred under reflux for 4 h. The solvent was removed and the residue was re-dissolved in methylene chloride (10 mL) and extracted with water (3 × 10 mL). An organic phase was dried (MgSO_4_), concentrated, and the crude product was purified on a silica gel column with a methylene chloride:hexane (7:3, *v/v*) mixture followed by crystallization (chloroform-petroleum ether or ethyl acetate) to give pure quinazolinones.

*3-Benzyl-6-bromo-2-vinylquinazolin-4(3H)-one* (**13b**). A white amorphous solid, m.p. = 71–72 °C (re-crystallized from ethyl acetate). IR (KBr, cm^−1^) ν_max_: 3097, 3036, 2926, 1608, 1571, 1553, 1470, 1290, 1153, 1112, 989, 841, 678, 581, 437. ^1^H-NMR (200 MHz, CDCl_3_): δ = 8.30–8.27 (m, 1H), 7.88–7.82 (m, 1H), 7.77–7.73 (m, 1H), 7.57–7.50 (m, 2H), 7.48–7.37 (m, 3H), 6.91 (dd, ^3^*J* = 17.2 Hz, ^3^*J* = 9.9 Hz, 1H, C*H*=CH_2_), 6.73 (dd, ^3^*J* = 17.2 Hz, ^2^*J* = 2.5 Hz, 1H, CH=C*H*_2_), 5.82 (dd, ^3^*J* = 9.9 Hz, ^2^*J* = 2.5 Hz, 1H, CH=C*H*_2_), 5.67 (s, 2H, N-C*H*_2_). ^13^C-NMR (151 MHz, CDCl_3_): δ = 165.16 (C=O), 160.28, 150.36, 137.00, 136.88, 136.02, 129.45, 128.67, 128.45, 126.09, 124.36, 120.03, 116.67, 68.69 (N-CH_2_). Anal. Calcd. for C_17_H_13_BrN_2_O: C, 59.84; H, 3.84; N, 8.21. Found: C, 59.92; H, 3.68; N, 8.29.

*6-Bromo-3-(2-nitrobenzyl)-2-vinylquinazolin-4(3H)-one* (**13c**). A yellowish amorphous solid, m.p. 117–119 °C (re-crystallized from chloroform-petroleum ether). IR (KBr, cm^−1^) ν_max_: 3078, 2980, 2925, 2854, 1689, 1613, 1573, 1526, 1489, 1390, 1354, 1287, 1242, 1116, 1053, 1024, 968, 836, 791, 730. ^1^H-NMR (600 MHz, CDCl_3_): δ = 8.33 (d, ^4^*J* = 2.2 Hz, 1H, *H*C5), 8.18–8.16 (m, 1H), 7.90 (dd, ^3^*J* = 8.9 Hz, ^4^*J* = 2.2 Hz, 1H, *H*C7), 7.81 (d, ^3^*J* = 8.9 Hz, 1H, *H*C8), 7.80–7.78 (m, 1H), 7.71–7.69 (m, 1H), 7.56–7.53 (m, 1H), 6.87 (dd, ^3^*J* = 17.2 Hz, ^3^*J* = 10.4 Hz, 1H, C*H*=CH_2_), 6.64 (dd, ^3^*J* = 17.2 Hz, ^2^*J* = 1.5 Hz, 1H, CH=C*H*_2_), 6.10 (s, 2H, N-C*H*_2_), 5.80 (dd, ^3^*J* = 10.4 Hz, ^2^*J* = 1.5 Hz, 1H, CH=C*H*_2_). ^13^C-NMR (151 MHz, CDCl_3_): δ = 164.62 (C=O), 160.12, 150.32, 147.84, 137.31, 136.31, 133.78, 132.33, 129.51, 129.05, 128.87, 125.76, 125.07, 124.92, 120.38, 116.31, 65.14 (N-CH_2_). Anal. Calcd. for C_17_H_12_BrN_3_O_3_: C, 52.87; H, 3.13; N, 10.88. Found: C, 52.50; H, 2.80; N, 10.80.

*6-Bromo-3-(3-nitrobenzyl)-2-vinylquinazolin-4(3H)-one* (**13d**). A yellowish amorphous solid, m.p. 147–149 °C (re-crystallized from chloroform-petroleum ether). IR (KBr, cm^−1^) ν_max_: 3112, 3071, 2924, 2854, 1610, 1573, 1529, 1456, 1382, 1294, 1119, 836, 668. ^1^H-NMR (600 MHz, CDCl_3_): δ = 8.44 (brs, 1H), 8.32–8.30 (m, 1H), 8.26–8.24 (m, 1H), 7.91–7.88 (m, 2H), 7.80–7.78 (m, 1H), 7.64–7.61 (m, 1H), 6.91 (dd, ^3^*J* = 17.2 Hz, ^3^*J* = 10.4 Hz, 1H, C*H*=CH_2_), 6.73 (dd, ^3^*J* = 17.2 Hz, ^2^*J* = 1.4 Hz, 1H, CH=C*H*_2_), 5.84 (dd, ^3^*J* = 10.4 Hz, ^2^*J* = 1.4 Hz, 1H, CH=C*H*_2_), 5.78 (s, 2H, N-C*H*_2_). ^13^C-NMR (151 MHz, CDCl_3_): δ = 164.69 (C=O), 160.05, 150.49, 148.51, 138.14, 137.28, 136.68, 134.10, 129.73, 129.60, 125.84, 124.47, 123.37, 123.27, 120.35, 116.34, 67.22 (N-CH_2_). Anal. Calcd. for C_17_H_12_BrN_3_O_3_: C, 52.87; H, 3.13; N, 10.88. Found: C, 52.81; H, 2.98; N, 10.60.

*6-Bromo-3-(4-nitrobenzyl)-2-vinylquinazolin-4(3H)-one* (**13e**). Off-white amorphous solid, m.p. 121–123 °C (re-crystallized from chloroform-petroleum ether). IR (KBr, cm^−1^) ν_max_: 3418, 2924, 2853, 1739, 1608, 1573, 1524, 1343, 1292, 1117, 945, 833, 661. ^1^H-NMR (200 MHz, CDCl_3_): δ = 8.30–8.26 (m, 3H), 7.96–7.92 (m, 1H), 7.81–7.76 (m, 1H), 7.73–7.67 (m, 2H), 6.88 (dd, ^3^*J* = 17.2 Hz, ^3^*J* = 10.1 Hz, 1H, C*H*=CH_2_), 6.65 (dd, ^3^*J* = 17.2 Hz, ^2^*J* = 2.1 Hz, 1H, CH=C*H*_2_), 5.80 (dd, ^3^*J* = 10.1 Hz, ^2^*J* = 2.1 Hz, 1H, CH=C*H*_2_), 5.77 (s, 2H, N-C*H*_2_). ^13^C-NMR (151 MHz, CDCl_3_): δ = 164.69 (C=O), 160.03, 150.41, 147.93, 143.27, 137.37, 136.59, 129.58, 128.50, 125.80, 124.58, 123.93, 120.44, 116.33, 67.14 (N-CH_2_). Anal. Calcd. for C_17_H_12_BrN_3_O_3_: C, 52.87; H, 3.13; N, 10.88. Found: C, 52.81; H, 2.98; N, 10.60.

*6-Bromo-3-(2-fluorobenzyl)-2-vinylquinazolin-4(3H)-one* (**13f**). A white amorphous solid, m.p. 91–92 °C (re-crystallized from ethyl acetate). IR (KBr, cm^−1^) ν_max_: 3078, 2967, 1568, 1555, 1486, 1420, 1348, 1293, 1238, 1115, 938, 828, 760, 675. ^1^H-NMR (600 MHz, CDCl_3_): δ = 8.31 (d, ^4^*J* = 2.2 Hz, 1H, *H*C5), 7.89 (dd, ^3^*J* = 8.9 Hz, ^4^*J* = 2.2 Hz, 1H, *H*C7), 7.68 (d, ^3^*J* = 8.9 Hz, 1H, *H*C8), 7.61–7.58 (m, 1H), 7.42–7.38 (m, 1H), 7.23–7.20 (m, 1H), 7.18–7.15 (m, 1H), 6.92 (dd, ^3^*J* = 17.2 Hz, ^3^*J* = 10.4 Hz, 1H, C*H*=CH_2_), 6.78 (dd, ^3^*J* = 17.2 Hz, ^2^*J* = 1.7 Hz, 1H, CH=C*H*_2_), 5.84 (dd, ^3^*J* = 10.4 Hz, ^2^*J* = 1.7 Hz, 1H, CH=C*H*_2_), 5.78 (s, 2H, N-C*H*_2_). ^13^C-NMR (151 MHz, CDCl_3_): δ = 164.99 (C=O), 161.20 (d, ^1^*J*_(CF)_ = 248.8 Hz, C2′), 160.24, 150.39, 137.03, 136.81, 130.70 (d, ^3^*J*_(CCCF)_ = 4.0 Hz, C4′), 130.39 (d, ^3^*J*_(CCCF)_ = 7.9 Hz, C6′), 129.47, 126.04, 124.44, 124.25 (d, ^4^*J*_(CCCCF)_ = 3.4 Hz, C5′), 123.24 (d, ^2^*J*_(CCF)_ = 14.5 Hz, C3′), 120.06, 116.59, 115.63 (d, ^2^*J*_(CCF)_ = 21.5 Hz, C1′), 62.51 (d, ^3^*J*_(CCF)_ = 4.4 Hz, N-CH_2_). Anal. Calcd. for C_17_H_12_BrFN_2_O: C, 56.85; H, 3.37; N, 7.80. Found: C, 56.62; H, 2.99; N, 7.79.

*6-Bromo-3-(3-fluorobenzyl)-2-vinylquinazolin-4(3H)-one* (**13g**). A yellowish amorphous solid, m.p. 78–80 °C (re-crystallized from ethyl acetate). IR (KBr, cm^−1^) ν_max_: 3070, 2980, 2925, 2853, 1614, 1568, 1490, 1416, 1336, 1286, 1115, 1055, 967, 836, 790. ^1^H-NMR (600 MHz, CDCl_3_): δ = 8.32 (d, ^4^*J* = 2.2 Hz, 1H, *H*C5), 7.89 (dd, ^3^*J* = 8.9 Hz, ^4^*J* = 2.2 Hz, 1H, *H*C7), 7.80 (d, ^3^*J* = 8.9 Hz, 1H, *H*C8), 7.43–7.39 (m, 1H), 7.34–7.32 (m, 1H), 7.28–7.26 (m, 1H), 7.11–7.08 (m, 1H), 6.92 (dd, ^3^*J* = 17.1 Hz, ^3^*J* = 10.4 Hz, 1H, C*H*=CH_2_), 6.73 (dd, ^3^*J* = 17.1 Hz, ^2^*J* = 1.7 Hz, 1H, CH=C*H*_2_), 5.84 (dd, ^3^*J* = 10.4 Hz, ^2^*J* = 1.7 Hz, 1H, CH=C*H*_2_), 5.69 (s, 2H, N-C*H*_2_). ^13^C-NMR (151 MHz, CDCl_3_): δ = 164.95 (C=O), 162.95(d, ^1^*J*_(CF)_ = 246.6 Hz, C3′), 160.19, 150.43, 138.50 (d, ^3^*J*_(CCCF)_ = 7.5 Hz, C5′), 137.13, 136.79, 130.25 (d, *J* = 8.1 Hz, C1′), 129.53, 125.98, 124.42, 123.74 (d, ^4^*J*_(CCCCF)_ = 3.1 Hz, C6′), 120.18, 116.54, 115.33 (d, ^2^*J*_(CCF)_ = 21.0 Hz, C4′), 115.14 (d, ^2^*J*_(CCF)_ = 22.0 Hz, C2′), 67.77 (d, ^4^*J*_CCCCF_ = 1.5 Hz, N-CH_2_). Anal. Calcd. for C_17_H_12_BrFN_2_O: C, 56.85; H, 3.37; N, 7.80. Found: C, 56.99; H, 2.97; N, 7.84.

*6-Bromo-3-(4-fluorobenzyl)-2-vinylquinazolin-4(3H)-one* (**13h**). A yellowish amorphous solid, m.p. 100–103 °C (re-crystallized from chloroform-petroleum ether). IR (KBr, cm^−1^) ν_max_: 3064, 2926, 1608, 1565, 1510, 1487, 1354, 1219, 1118, 989, 832, 697, 501. ^1^H-NMR (200 MHz, CDCl_3_): δ = 8.26 (d, ^4^*J* = 2.1 Hz, 1H, *H*C5), 7.85 (dd, ^3^*J* = 8.9 Hz, ^4^*J* = 2.1 Hz, 1H, *H*C7), 7.75 (d, ^3^*J* = 8.9 Hz, 1H, *H*C8), 7.55–7.48 (m, 2H), 7.15–7.06 (m, 2H), 6.90 (dd, ^3^*J* = 17.2 Hz, ^3^*J* = 11.5 Hz, 1H, C*H*=CH_2_), 6.72 (dd, ^3^*J* = 17.2 Hz, ^2^*J* = 2.3 Hz, 1H, CH=C*H*_2_), 5.82 (dd, ^3^*J* = 11.5 Hz, ^2^*J* = 2.3 Hz, 1H, CH=C*H*_2_), 5.63 (s, 2H, N-C*H*_2_). ^13^C-NMR (151 MHz, CDCl_3_): δ = 165.04 (C=O), 162.82 (d, ^1^*J*_(CF)_ = 247.1 Hz, C4′), 160.20, 150.40, 137.04, 136.88, 131.84 (d, ^4^*J*_(CCCCF)_ = 3.1 Hz, C1′), 130.39 (d, ^3^*J*_(CCCF)_ = 8.6 Hz, C2′, C6′), 129.50, 125.99, 124.28, 120. 08, 116.60, 115.62 (d, ^2^*J*_(CCF)_ = 21.9 Hz, C3′, C5′), 67.94 (N-CH_2_). Anal. Calcd. for C_17_H_12_BrFN_2_O: C, 56.85; H, 3.37; N, 7.80. Found: C, 56.50; H, 3.01; N, 7.76.

*6-Bromo-3-(2,4-difluorobenzyl)-2-vinylquinazolin-4(3H)-one* (**13i**). A yellowish amorphous solid, m.p. 88–90 °C (re-crystallized from chloroform-petroleum ether). IR (KBr, cm^−1^) ν_max_: 3079, 2923, 2852, 1614, 1565, 1508, 1488, 1416, 1349, 1279, 1099, 955, 834, 728, 538. ^1^H-NMR (600 MHz, CDCl_3_): δ = 8.27 (d, ^4^*J* = 2.1 Hz, 1H, *H*C5), 7.88 (dd, ^3^*J* = 8.9 Hz, ^4^*J* = 2.1 Hz, 1H, *H*C7), 7.78 (d, ^3^*J* = 8.9 Hz, 1H, *H*C8), 7.59–7.55 (m, 1H), 6.96–6.89 (m, 2H), 6.89 (dd, ^3^*J* = 17.2 Hz, ^3^*J* = 10.0 Hz, 1H, C*H*=CH_2_), 6.72 (dd, ^3^*J* = 17.2 Hz, ^2^*J* = 2.4 Hz, 1H, CH=C*H*_2_), 5.82 (dd, ^3^*J* = 10.0 Hz, ^2^*J* = 2.4 Hz, 1H, CH=C*H*_2_), 5.68 (s, 2H, N-C*H*_2_). ^13^C-NMR (151 MHz, CDCl_3_) δ: 164.86 (C=O), 163.23 (dd, ^1^*J*_(CF)_ = 250.4 Hz, ^3^*J*_(CCCF)_ = 12.0 Hz, C2′), 161.45 (dd, ^1^*J*_(CF)_ = 251.8 Hz, ^3^*J*_(CCCF)_ = 12.1 Hz, C4′), 160.15, 150.38, 137.06, 136.80, 131.83 (dd, ^3^*J*_(CCCF)_ = 9.8 Hz, ^3^*J*_(CCCF)_ = 5.4 Hz, C6′), 129.49, 125.95, 124.39, 120.12, 119.29 (dd, ^2^*J*_(CCF)_ = 15.0 Hz, ^4^*J*_(CCCCF)_ = 4.0 Hz, C1′), 116.48, 111.50 (dd, ^2^*J*_(CCF)_ = 21.7 Hz, ^4^*J*_(CCCCF)_ = 4.1 Hz, C5′), 104.16 (dd, ^2^*J*_(CCF)_ = 25.3 Hz, ^2^*J*_(CCF)_ = 25.3 Hz, C3′), 61.93 (d, ^3^*J*_(CCCF)_ = 3.4 Hz, N-CH_2_) Anal. Calcd. for C_17_H_11_BrF_2_N_2_O: C, 54.13; H, 2.94; N, 7.43. Found: C, 54.42; H, 3.14; N, 7.04.

### 3.4. General Procedure for the Synthesis of N3-Alkylated 6-bromo-2-vinyl-3H-quinazolin-4-ones ***13j*** and ***13k***

To the solution of 6-bromo-2-vinyl-3*H*-quinazolin-4-one (0.251 g, 1.00 mmol) in acetonitrile (15 mL) potassium hydroxide (0.168 g, 3.00 mmol) was added. After 15 min iodomethane (0.124 mL, 2.00 mmol) or iodoethane (0.088 mL, 1.10 mmol) was added and the reaction mixture was stirred at 60 °C for 5 h. The solvent was removed and the residue was re-dissolved in methylene chloride (10 mL) and extracted with water (3 × 10 mL). The organic layer was dried (MgSO_4_), concentrated, and the crude product was purified on a silica gel column with a methylene chloride:hexane mixture (7:3, *v/v*) mixture followed by crystallization (chloroform:petroleum ether).

*6-Bromo-3-methyl-2-vinylquinazolin-4(3H)-one* (**13j**). A white amorphous solid, m.p. = 147–148 °C (re-crystallized from chloroform-petroleum ether). IR (KBr, cm^−1^) ν_max_: 2956, 2924, 1675, 1661, 1555, 1468, 1337, 1260, 1025, 981, 791, 645. ^1^H-NMR (200 MHz, CDCl_3_): δ = 8.21 (d, ^4^*J* = 2.1 Hz, 1H, *H*C5), 7.79 (dd, ^3^*J* = 8.9 Hz, ^4^*J* = 2.1 Hz, 1H, *H*C7), 7.67 (d, ^3^*J* = 8.9 Hz, 1H, *H*C8), 6.83 (dd, ^3^*J* = 17.2 Hz, ^3^*J* = 9.9 Hz, 1H, C*H*=CH_2_), 6.66 (dd, ^3^*J* = 17.2 Hz, ^2^*J* = 2.5 Hz, 1H, CH=C*H*_2_), 5.74 (dd, ^3^*J* = 9.9 Hz, ^2^*J* = 2.5 Hz, 1H, CH=C*H*_2_), 4.14 (s, 3H, C*H*_3_). ^13^C-NMR (151 MHz, CDCl_3_): δ = 161.06 (C=O), 152.56, 146.20, 137.33, 129.34, 129.29, 128.92, 127.19, 121.95, 120.12, 30.95. Anal. Calcd. for C_11_H_9_BrN_2_O: C, 49.84; H, 3.42; N, 10.57. Found: C, 50.11; H, 3.42; N, 10.56.

*6-Bromo-3-ethyl-2-vinylquinazolin-4(3H)-one* (**13k**). A white amorphous solid, m.p. = 62–64 °C (re-crystallized from chloroform-petroleum ether). IR (KBr, cm^−1^) ν_max_: 3097, 3035, 2956, 2926, 2853, 1610, 1571, 1554, 1488, 1428, 1347, 1291, 1153, 1018, 842, 807, 678. ^1^H-NMR (200 MHz, CDCl_3_): δ = 8.22 (d, ^4^*J* = 2.2 Hz, 1H, *H*C5), 7.79 (dd, ^3^*J* = 8.9 Hz, ^4^*J* = 2.2 Hz, 1H, *H*C7), 7.68 (d, ^3^*J* = 8.9 Hz, 1H, *H*C8), 6.82 (dd, ^3^*J* = 17.2 Hz, ^3^*J* = 10.0 Hz, 1H, C*H*=CH_2_), 6.63 (dd, ^3^*J* = 17.2 Hz, ^2^*J* = 2.4 Hz, 1H, CH=C*H*_2_), 5.73 (dd, ^3^*J* = 10.0 Hz, ^2^*J* = 2.4 Hz, 1H, CH=C*H*_2_), 4.62 (q, ^3^*J* = 7.1 Hz, 2H, CH_3_C*H*_2_), 1.47 (t, ^3^*J* = 7.1 Hz, 3H, C*H*_3_CH_2_). ^13^C-NMR (151 MHz, CDCl_3_): δ = 161.06 (C=O), 160.37, 150.12, 136.90, 136.78, 129.34, 126.10, 124.19, 119.83, 116.71, 63.10, 14.28. Anal. Calcd. for C_12_H_11_BrN_2_O: C, 51.63; H, 3.97; N, 10.04. Found: C, 51.94; H, 3.75; N, 9.94.

### 3.5. General Procedure for the Synthesis of Isoxazolidines trans-***11*** and cis-***11***

A solution of the nitrone (0.195 g, 1.00 mmol) and the respective 6-bromo-2-vinylquinazolin-4(3*H*)-one (1.00 mmol) in toluene (2 mL) was stirred at 70 °C until the disappearance (TLC) of the starting nitrone. Solvents were evaporated in vacuo and crude products were subjected to chromatography on silica gel columns with chloroform:methanol (100:1, 50:1, 20:1, *v/v*) mixtures.

*Diethyl trans-[5-(6-bromo-4-oxo-3,4-dihydroquinazolin-2-yl)-2-methylisoxazolidin-3-yl]phosphonate* (*trans-***11a**). Colorless oil. IR (film, cm^−1^) ν_max_: 3316, 3171, 3090, 2980, 2974, 2783, 1660, 1625, 1486, 1412, 1301, 1234, 1054, 968, 834, 775, 575. ^1^H-NMR (600 MHz, CDCl_3_): δ = 10.63 (s, 1H, NH), 8.44 (d, ^4^*J* = 2.3 Hz, 1H, *H*C5′), 7.84 (dd, ^3^*J* = 8.6 Hz, ^4^*J* = 2.3 Hz, 1H, *H*C7′), 7.54 (d, ^3^*J* = 8.6 Hz, 1H, *H*C8′), 5.02 (dd, ^3^*J*_(H5–H4β)_ = 8.4 Hz, ^3^*J*_(H5–H4α)_ = 6.2 Hz, 1H, *H*C5), 4.30–4.21 (m, 4H, 2 × C*H*_2_OP), 3.25–3.21 (m, 1H, *H*C3), 3.04 (s, 3H, C*H*_3_N), 3.12 (dddd, ^3^*J*_(H4β–P)_ = 16.7 Hz, ^2^*J*_(H4β–H4α)_ = 12.9 Hz, ^3^*J*_(H4β–H5)_ = 8.6 Hz, ^3^*J*_(H4β–H3)_ = 8.3 Hz, 1H, *H*_β_C4), 2.94 (dddd, ^3^*J*_(H4α–P)_ = 12.9 Hz, ^2^*J*_(H4α–H4β)_ = 12.9 Hz, ^3^*J*_(H4α–H3)_ = 10.0 Hz, ^3^*J*_(H4α–H5)_ = 6.2 Hz, 1H, *H*_α_C4), 1.40 (t, ^3^*J* = 7.1 Hz, 3H, C*H*_3_CH_2_OP), 1.38 (t, ^3^*J* = 7.0 Hz, 3H, C*H*_3_CH_2_OP). ^13^C-NMR (151 MHz, CDCl_3_): δ = 160.30 (C=O), 156.50, 147.35, 137.66, 129.26, 128.96, 123.27, 120.46, 74.68 (d, ^3^*J*_(CCCP)_ = 8.6 Hz, C5), 64.60 (d, ^1^*J*_(CP)_ = 168.1 Hz, C3), 63.51 (d, ^2^*J*_(COP)_ = 6.6 Hz, CH_2_OP), 62.61 (d, ^2^*J*_(COP)_ = 6.9 Hz, CH_2_OP), 46.07 (CH_3_N), 40.92 (C4), 16.55 (d, ^3^*J*_(CCOP)_ = 6.2 Hz, *C*H_3_CH_2_OP), 16.48 (d, ^3^*J*_(CCOP)_ = 5.5 Hz, *C*H_3_CH_2_OP). ^31^P-NMR (243 MHz, CDCl_3_): δ = 20.51. Anal. Calcd. for C_16_H_21_BrN_3_O_5_P: C, 43.07; H, 4.74; N, 9.42. Found: C, 43.09; H, 4.52; N, 9.35.

*Diethyl cis-[5-(6-bromo-4-oxo-3,4-dihydroquinazolin-2-yl)-2-methylisoxazolidin-3-yl]phosphonate* (*cis*-**11a**). Colorless oil. IR (film, cm^−1^) ν_max_: 3090, 2959, 2925, 2865, 1661, 1626, 1601, 1461, 1336, 1234, 1054, 969, 834, 575. ^1^H-NMR (600 MHz, CDCl_3_): δ = 10.63 (s, 1H, NH), 8.44 (d, ^4^*J* = 2.3 Hz, 1H, *H*C5′), 7.84 (dd, ^3^*J* = 8.6 Hz, ^4^*J* = 2.3 Hz, 1H, *H*C7′), 7.54 (d, ^3^*J* = 8.6 Hz, 1H, *H*C8′), 5.07 (dd, ^3^*J*_(H5–H4α)_ = 9.2 Hz, ^3^*J*_(H5–H4β)_ = 4.3 Hz, 1H, *H*C5), 4.23–4.12 (m, 4H, 2 × C*H*_2_OP), 3.21 (dddd, ^3^*J*_(H4β–P)_ = 19.8 Hz, ^2^*J*_(H4β–H4α)_ = 10.9 Hz, ^3^*J*_(H4β–H3)_ = 6.8 Hz, ^3^*J*_(H4β–H5)_ = 4.3 Hz, 1H, *H*_β_C4), 3.12 (ddd, ^3^*J*_(H3–H4β)_ = 6.8 Hz, ^3^*J*_(H3–H4α)_ = 9.6 Hz, ^2^*J*_(H3–P)_ = 4.4 Hz 1H, *H*C3), 3.00 (s, 3H, C*H*_3_N), 2.83 (dddd, ^3^*J*_(H4α–P)_ = 13.1 Hz, ^2^*J*_(H4α–H4β)_ = 10.9 Hz, ^3^*J*_(H4α–H3)_ = 9.6 Hz, ^3^*J*_(H4α–H5)_ = 9.2 Hz, 1H, *H*_α_C4), 1.32 (t, ^3^*J* = 7.0 Hz, 3H, C*H*_3_CH_2_OP), 1.27 (t, ^3^*J* = 7.1 Hz, 3H, C*H*_3_CH_2_OP). ^13^C-NMR (151 MHz, CDCl_3_): δ = 160.33 (C=O), 156.50, 147.35, 137.65, 129.26, 128.96, 123.27, 120.46, 75.58 (d, ^3^*J*_(CCCP)_ = 6.6 Hz, C5), 63.58 (d, ^1^*J*_(CP)_ = 169.1 Hz, C3), 63.18 (d, ^2^*J*_(COP)_ = 6.5 Hz, CH_2_OP), 63.15 (d, ^2^*J*_(COP)_ = 6.4 Hz, CH_2_OP), 45.58 (d, ^3^*J*_(CNCP)_ = 5.8 Hz, CH_3_N), 37.71 (C4), 16.38 (d, ^3^*J*_(CCOP)_ = 5.3 Hz, *C*H_3_CH_2_OP), 16.37 (d, ^3^*J*_(CCOP)_ = 5.4 Hz, *C*H_3_CH_2_OP). ^31^P-NMR (243 MHz, CDCl_3_): δ = 20.92. Anal. Calcd. for C_16_H_21_BrN_3_O_5_P: C, 43.07; H, 4.74; N, 9.42. Found: C, 43.12; H, 4.35; N, 9.33.

*Diethyl trans-[5-(3-benzyl-6-bromo-4-oxo-3,4-dihydroquinazolin-2-yl)-2-methylisoxazolidin-3-yl]phosphonate* (*trans*-**11b**). A yellowish oil. IR (film, cm^−1^) ν_max_: 3040, 2980, 2926, 2853, 1613, 1568, 1490, 1418, 1353, 1239, 1056, 1025, 835, 822, 699. ^1^H-NMR (600 MHz, CDCl_3_): δ = 8.33 (d, ^4^*J* = 2.3 Hz, 1H, *H*C5′), 7.90 (dd, ^3^*J* = 8.9 Hz, ^4^*J* = 2.3 Hz, 1H, *H*C7′), 7.83 (d, ^3^*J* = 8.9 Hz, 1H, *H*C8′), 7.55–7.53 (m, 2H), 7.45–7.42 (m, 2H), 7.40–7.37 (m, 1H), 5.66 (s, 2H, N-C*H*_2_), 5.25 (dd, ^3^*J*_(H5–H4β)_ = 6.8 Hz, ^3^*J*_(H5–H4α)_ = 6.4 Hz, 1H, *H*C5), 4.33–4.19 (m, 4H, 2 × C*H*_2_OP), 3.42–3.39 (m, 1H, *H*C3), 3.07–2.94 (m, 2H, *H*_α_C4, *H*_β_C4), 3.05 (s, 3H, C*H*_3_N), 1.42 (t, ^3^*J* = 7.0 Hz, 3H, C*H*_3_CH_2_OP), 1.39 (t, ^3^*J* = 6.9 Hz, 3H, C*H*_3_CH_2_OP). ^13^C-NMR (151 MHz, CDCl_3_): δ = 166.08 (C=O), 163.35, 149.91, 137.20, 135.69, 129.67, 128.67, 128.65, 128.52, 128.45, 125.97, 120.69, 80.15 (d, ^3^*J*_(CCCP)_ = 8.5 Hz, C5), 69.14 (N-CH_2_), 64.41 (d, ^1^*J*_(CP)_ = 168.8 Hz, C3), 63.26 (d, ^2^*J*_(COP)_ = 6.5 Hz, CH_2_OP), 62.42 (d, ^2^*J*_(COP)_ = 7.2 Hz, CH_2_OP), 46.62 (CH_3_N), 37.90 (C4), 16.58 (d, ^3^*J*_(CCOP)_ = 5.6 Hz, *C*H_3_CH_2_OP), 16.52 (d, ^3^*J*_(CCOP)_ = 5.6 Hz, *C*H_3_CH_2_OP). ^31^P-NMR (243 MHz, CDCl_3_): δ = 22.04. Anal. Calcd. for C_23_H_27_BrN_3_O_5_P: C, 51.50; H, 5.07; N, 7.83. Found: C, 51.78; H, 5.11; N, 7.70.

*Diethyl trans-{5-[6-bromo-3-(2-nitrobenzyl)-4-oxo-3,4-dihydroquinazolin-2-yl]-2-methylisoxazolidin-3-yl}phosphonate* (*trans*-**11c**). A yellowish oil. IR (film, cm^−1^) ν_max_: 3444, 3077, 2979, 2925, 2853, 1690, 1614, 1575, 1527, 1489, 1413, 1355, 1337, 1055, 1024, 835, 791, 730, 574. ^1^H-NMR (600 MHz, CDCl_3_): δ = 8.36 (d, ^4^*J* = 2.0 Hz, 1H, *H*C5′), 8.20–8.18 (m, 1H), 7.95 (dd, ^3^*J* = 8.9 Hz, ^4^*J* = 2.3 Hz, 1H, *H*C7′), 7.87 (d, ^4^*J* = 8.9 Hz, 1H, *H*C8′), 7.78–7.76 (m, 1H), 7.72–7.69 (m, 1H), 7.57–7.55 (m, 1H), 6.10 (AB, *J*_AB_ = 14.6 Hz, 1H, N-C*H*_2b_), 6.07 (AB, *J*_AB_ = 14.6 Hz, 1H, N-C*H*_2a_), 5.21 (dd, ^3^*J*_(H5–H4β)_ = 8.0 Hz, ^3^*J*_(H5–H4α)_ = 6.1 Hz, 1H, *H*C5), 4.31–4.22 (m, 4H, 2 × C*H*_2_OP), 3.36–3.33 (m, 1H, *H*C3), 3.00 (s, 3H, C*H*_3_N), 2.99 (dddd, ^2^*J*_(H4β–P)_ = 16.6 Hz, ^3^*J*_(H4β–H4α)_ = 12.6 Hz, ^3^*J*_(H4β–H3)_ = 8.2 Hz, ^3^*J*_(H4β–H5)_ = 8.0 Hz, 1H, *H*_β_C4), 2.38 (dddd, ^2^*J*_(H4α–H4β)_ = 12.6 Hz, ^3^*J*_(H4α–P)_ = 10.2 Hz, ^3^*J*_(H4α–H3)_ = 8.8 Hz, ^3^*J*_(H4α–H5)_ = 6.1 Hz, 1H, *H*_α_C4), 1.41 (t, ^3^*J* = 7.1 Hz, 3H, C*H*_3_CH_2_OP), 1.39 (t, ^3^*J* = 7.1 Hz, 3H, C*H*_3_CH_2_OP). ^13^C-NMR (151 MHz, CDCl_3_): δ = 165.56 (C=O), 163.42, 150.09, 147.81, 137.49, 133.80, 132.03, 129.85, 129.14, 129.00, 125.66, 125.15, 121.03, 116.40, 80.08 (d, ^3^*J*_(CCCP)_ = 7.8 Hz, C5), 65.64 (N-*C*H_2_), 64.30 (d, ^1^*J*_(CP)_ = 168.5 Hz, C3), 63.21 (d, ^2^*J*_(COP)_ = 6.3 Hz, CH_2_OP), 62.49 (d, ^2^*J*_(COP)_ = 6.8 Hz, CH_2_OP), 46.61 (d, ^3^*J*_(CNCP)_ = 4.2 Hz, CH_3_N), 37.91 (C4), 16.54 (d, ^3^*J*_(CCOP)_ = 5.6 Hz, *C*H_3_CH_2_OP), 16.49 (d, ^3^*J*_(CCOP)_ = 5.5 Hz, *C*H_3_CH_2_OP). ^31^P-NMR (243 MHz, CDCl_3_): δ = 21.85. Anal. Calcd. for C_23_H_26_BrN_4_O_7_P: C, 47.52; H, 4.51; N, 9.64. Found: C, 47.22; H, 4.40; N, 9.35.

*Diethyl trans-{5-[6-bromo-3-(3-nitrobenzyl)-4-oxo-3,4-dihydroquinazolin-2-yl]-2-methylisoxazolidin-3-yl}phosphonate* (*trans*-**11d**). A yellowish oil. IR (film, cm^−1^) ν_max_: 3078, 2980, 2926, 2854, 1613, 1566, 1531, 1489, 1416, 1348, 1242, 1115, 1053, 1024, 966, 836, 805, 733, 671. ^1^H-NMR (600 MHz, CDCl_3_): δ = 8.44–8.42 (m, 1H), 8.32 (d, ^4^*J* = 2.0 Hz, 1H, *H*C5′), 8.24–8.22 (m, 1H), 7.92 (dd, ^3^*J* = 8.9 Hz, ^4^*J* = 2.0 Hz, 1H, *H*C7′), 7.90–7.88 (m, 1H), 7.84 (d, ^3^*J* = 8.9 Hz, 1H, *H*C8′), 7.63–7.60 (m, 1H), 5.77 (s, 2H, N-C*H*_2_), 5.29 (dd, ^3^*J*_(H5–H4β)_ = 8.0 Hz, ^3^*J*_(H5–H4α)_ = 6.4 Hz, 1H, *H*C5), 4.34–4.23 (m, 4H, 2 × C*H*_2_OP), 3.44–3.37 (m, 1H, *H*C3), 3.10–2.94 (m, 2H, *H*_α_C4, *H*_β_C4), 3.07 (s, 3H, C*H*_3_N), 1.43 (t, ^3^*J* = 7.1 Hz, 3H, C*H*_3_CH_2_OP), 1.41 (t, ^3^*J* = 7.0 Hz, 3H, C*H*_3_CH_2_OP). ^13^C-NMR (151 MHz, CDCl_3_): δ = 165.59 (C=O), 163.05, 150.03, 148.49, 137.81, 137.49, 134.31, 129.78, 129.76, 125.74, 123.45, 123.29, 121.03, 116.42, 80.00 (d, ^3^*J*_(CCCP)_ = 8.4 Hz, C5), 65.61 (N-CH_2_), 64.38 (d, ^1^*J*_(CP)_ = 168.5 Hz, C3), 63.26 (d, ^2^*J*_(COP)_ = 6.5 Hz, CH_2_OP), 62.50 (d, ^2^*J*_(COP)_ = 6.8 Hz, CH_2_OP), 46.57 (CH_3_N), 37.77 (C4), 16.56 (d, ^3^*J*_(CCOP)_ = 5.6 Hz, *C*H_3_CH_2_OP), 16.50 (d, ^3^*J*_(CCOP)_ = 5.9 Hz, *C*H_3_CH_2_OP). ^31^P-NMR (243 MHz, CDCl_3_): δ = 21.52. Anal. Calcd. for C_23_H_26_BrN_4_O_7_P: C, 47.52; H, 4.51; N, 9.64. Found: C, 47.71; H, 4.51; N, 9.44.

*Diethyl trans-{5-[6-bromo-3-(4-nitrobenzyl)-4-oxo-3,4-dihydroquinazolin-2-yl]-2-methylisoxazolidin-3-yl}phosphonate* (*trans*-**11e**). A yellowish oil. IR (film, cm^−1^) ν_max_: 3069, 2969, 2925, 2854, 1610, 1571, 1523, 1490, 1343, 1285, 1241, 1114, 1027, 968, 837. ^1^H-NMR (600 MHz, CDCl_3_): δ = 8.35 (d, ^4^*J* = 2.0 Hz, 1H, *H*C5′), 1H), 8.30–8.28 (m, 2H), 7.95 (dd, ^3^*J* = 8.9 Hz, ^4^*J* = 2.0 Hz, 1H, *H*C7′), 7.86 (d, ^3^*J* = 8.9 Hz, 1H, *H*C8′), 7.72–7.71 (m, 2H), 5.76 (s, 1H, N-C*H*_2_), 5.24 (dd, ^3^*J*_(H5–H4β)_ = 6.4 Hz, ^3^*J*_(H5–H4α)_ = 6.0 Hz, 1H, *H*C5), 4.32–4.22 (m, 4H, 2 × C*H*_2_OP), 3.37–3.33 (m, 1H, *H*C3), 3.07–2.98 (m, 1H, *H*_β_C4), 3.03 (s, 3H, C*H*_3_-N), 2.94 (dddd, ^3^*J*_(H4α–P)_ = 12.4 Hz, ^2^*J*_(H4α–H4β)_ = 12.4 Hz, ^3^*J*_(H4α–H3)_ = 9.2 Hz, ^3^*J*_(H4α–H5)_ = 6.0 Hz, 1H, *H*_α_C4), 1.41 (t, ^3^*J* = 7.0 Hz, 3H, C*H*_3_CH_2_OP), 1.39 (t, ^3^*J* = 7.0 Hz, 3H, C*H*_3_CH_2_OP). ^13^C-NMR (151 MHz, CDCl_3_): δ = 165.57 (C=O), 163.06, 150.04, 147.95, 142.93, 137.55, 129.83, 128.67, 125.69, 123.92, 121.09, 116.42, 79.97 (d, ^3^*J*_(CCCP)_ = 8.6 Hz, C5), 67.55 (s, N-CH_2_), 64.40 (d, ^1^*J*_(CP)_ = 168.3 Hz, C3), 63.28 (d, ^2^*J*_(COP)_ = 6.5 Hz, CH_2_OP), 62.46 (d, ^2^*J*_(COP)_ = 7.1 Hz, CH_2_OP), 46.55 (CH_3_N), 37.77 (C4), 16.56 (d, ^3^*J*_(CCOP)_ = 5.7 Hz, *C*H_3_CH_2_OP), 16.50 (d, ^3^*J*_(CCOP)_ = 5.8 Hz, *C*H_3_CH_2_OP). ^31^P-NMR (243 MHz, CDCl_3_): δ = 21.85. Anal. Calcd. for C_23_H_26_BrN_4_O_7_P: C, 47.52; H, 4.51; N, 9.64. Found: C, 47.75; H, 4.54; N, 9.39.

*Diethyl trans-{5-[6-bromo-3-(2-fluorobenzyl)-4-oxo-3,4-dihydroquinazolin-2-yl]-2-methylisoxazolidin-3-yl}phosphonate* (*trans*-**11f**). Data presented below were extracted from spectra of a 88:12 mixture of trans-**11f** and cis-**11f**. Yellowish oil. IR (film, cm^−1^) ν_max_: 3069, 2981, 2929, 2909, 1614, 1567, 1490, 1456, 1353, 1285, 1116, 1025, 964, 869, 760, 691. ^1^H-NMR (600 MHz, CDCl_3_): δ = 8.31 (d, ^4^*J* = 2.1 Hz, 1H, HC5′), 7.90 (dd, ^3^*J* = 8.9 Hz, ^4^*J* = 2.1 Hz, 1H, HC7′), 7.84 (d, ^3^*J* = 8.9 Hz, 1H, HC8′), 7.58–7.55 (m, 1H), 7.40–7.36 (m, 1H), 7.21–7.18 (m, 1H), 7.16–7.13 (m, 1H), 5.73 (AB, *J*_AB_ = 12.4 Hz, 1H, N-CH_2b_), 5.71 (AB, *J*_AB_ = 12.4 Hz, 1H, N-CH_2a_), 5.25 (dd, ^3^*J*_(H5–H4β)_ = 7.9 Hz, ^3^*J*_(H5–H4α)_ = 6.2 Hz, 1H, HC5), 4.33–4.18 (m, 4H, 2 × CH_2_OP), 3.42–3.39 (m, 1H, C3), 3.05 (s, 3H, CH_3_-N), 3.03–2.96 (m, 1H, H_β_C4), 2.96 (dddd, ^3^*J*_(H4α–P)_ = 12.5 Hz, ^2^*J*_(H4α–H4β)_ = 12.5 Hz, ^3^*J*_(H4α–H3)_ = 8.9 Hz, ^3^*J*_(H4α–H5)_ = 6.2 Hz, 1H, H_α_C4), 1.42 (t, ^3^*J* = 7.0 Hz, 3H, CH_3_CH_2_OP), 1.39 (t, ^3^*J* = 7.1 Hz, 3H, CH_3_CH_2_OP). ^13^C-NMR (151 MHz, CDCl_3_): δ = 165.94 (C=O), 163.31, 161.19 (d, ^1^*J*_(CF)_ = 248.8 Hz, C2″), 149.96, 137.25, 130.81 (d, ^3^*J*_(CCCF)_ = 3.5 Hz, C4″), 130.52 (d, ^3^*J*_(CCCF)_ = 8.0 Hz, C6″), 129.68, 125.94, 124.26 (d, ^4^*J*_(CCCCF)_ = 3.4 Hz, C5″), 122.93 (d, ^2^*J*_(CCF)_ = 14.5 Hz, C3″), 120.75, 116.65, 115.66 (d, ^2^*J*_(CCF)_ = 21.0 Hz, C1″), 80.14 (dd, ^3^*J*_(CCCP)_ = 8.1 Hz, C5), 64.42 (d, ^1^*J*_(CP)_ = 168.3 Hz, C3), 63.25 (d, ^2^*J*_(COP)_ = 6.5 Hz, CH_2_OP), 63.03 (d, ^3^*J* = 4.3 Hz, N-CH_2_), 62.43 (d, ^2^*J*_(COP)_ = 7.2 Hz, CH_2_OP), 46.59 (CH_3_N), 37.89 (C4), 16.55 (d, ^3^*J*_(CCOP)_ = 5.7 Hz, CH_3_CH_2_OP), 16.50 (d, ^3^*J*_(CCOP)_ = 5.8 Hz, CH_3_CH_2_OP). ^31^P-NMR (243 MHz, CDCl_3_): δ = 22.01. Anal. Calcd. for C_23_H_26_BrFN_3_O_5_P: C, 49.83; H, 4.73; N, 7.58. Found: C, 49.49; H, 4.53; N, 7.71 (obtained on a 88:12 mixture of trans-**11f** and cis-**11f**).

*Diethyl trans-{5-[6-bromo-3-(3-fluorobenzyl)-4-oxo-3,4-dihydroquinazolin-2-yl]-2-methylisoxazolidin-3-yl}phosphonate* (*trans*-**11g**). Yellowish oil. IR (film, cm^−1^) ν_max_: 3071, 2979, 2926, 2853, 1613, 1572, 1490, 1452, 1415, 1345, 1285, 1255, 1114, 1055, 1025, 966, 836, 789, 749. ^1^H-NMR (200 MHz, CDCl_3_): δ = 8.35–8.33 (m, 1H), 7.91–7.89 (m, 1H), 7.84–7.82 (m, 1H), 7.41–7.37 (m, 1H), 7.30–7.28 (m, 1H), 7.26–7.24 (m, 1H), 7.08–7.05 (m, 1H), 5.64 (s, 1H, N-CH_2_), 5.24 (dd, ^3^*J*_(H5–H4β)_ = 7.9 Hz, ^3^*J*_(H5–H4α)_ = 6.5 Hz, 1H, *H*C5), 4.33–4.17 (m, 4H, 2 × C*H*_2_OP), 3.39–3.36 (m, 1H, C3), 3.04 (s, 3H, C*H*_3_-N), 3.05–2.99 (m, 1H, *H*_β_C4), 2.95 (dddd, ^3^*J*_(H4α–P)_ = 12.7 Hz, ^2^*J*_(H4α–H4β)_ = 12.7 Hz, ^3^*J*_(H4α–H3)_ = 9.7 Hz, ^3^*J*_(H4α–H5)_ = 6.5 Hz, 1H, *H*_α_C4), 1.41 (t, ^3^*J* = 7.0 Hz, 3H, C*H*_3_CH_2_OP), 1.39 (t, ^3^*J* = 6.9 Hz, 3H, C*H*_3_CH_2_OP). ^13^C-NMR (151 MHz, CDCl_3_): δ = 165.84 (C=O), 163.22, 162.91, (d, ^1^*J*_(CF)_ = 246.7 Hz, C3′), 149.97, 138.18 (d, ^3^*J*_(CCCF)_ = 7.6 Hz, C5′), 137.31, 130.26 (d, *J* = 7.9 Hz, C1′), 129.72, 125.84, 123.80 (d, ^4^*J*_(CCCCF)_ = 2.4 Hz, C6′), 120.83, 116.59, 115.45 (d, ^2^*J*_(CCF)_ = 21.0 Hz, C4′), 115.21 (d, ^2^*J*_(CCF)_ = 22.0 Hz, C2′), 80.06 (dd, ^3^*J*_(CCCP)_ = 8.0 Hz, C5), 68.19 (d, ^3^*J* = 1.7 Hz, N-CH_2_), 64.42 (d, ^1^*J*_(CP)_ = 168.3 Hz, C3), 63.24 (d, ^2^*J*_(COP)_ = 6.4 Hz, CH_2_OP), 62.44 (d, ^2^*J*_(COP)_ = 7.3 Hz, CH_2_OP), 46.57 (d, ^3^*J*_(CNCP)_ = 3.7 Hz, CH_3_N), 37.84 (s, C4), 16.55 (d, ^3^*J*_(CCOP)_ = 5.6 Hz, *C*H_3_CH_2_OP), 16.49 (d, ^3^*J*_(CCOP)_ = 5.9 Hz, *C*H_3_CH_2_OP). ^31^P-NMR (121.5 MHz, CDCl_3_): δ = 21.97. Anal. Cald. for C_23_H_26_BrFN_3_O_5_P: C, 49.83; H, 4.73; N, 7.58. Found: C, 49.49; H, 4.53; N, 7.71.

*Diethyl trans{5-[6-bromo-3-(4-fluorobenzyl)-4-oxo-3,4-dihydroquinazolin-2-yl]-2-methylisoxazolidin-3-yl}phosphonate* (*trans*-**11h**). A yellowish oil. IR (film, cm^−^^1^) ν_max_: 3072, 2981, 2926, 2853, 1611, 1568, 1512, 1490, 1430, 1351, 1285, 1227, 1160, 1114, 1099,1056, 1026, 965, 835.^1^H-NMR (600 MHz, CDCl_3_): δ = 8.31 (d, ^4^*J* = 2.2 Hz, 1H, *H*C5′), 7.91 (dd, ^3^*J* = 8.9 Hz, ^4^*J* = 2.2 Hz, 1H, *H*C7′), 7.84 (d, ^3^*J* = 8.9 Hz, 1H, *H*C8′), 7.55–7.53 (m, 2H), 7.14–7.11 (m, 2H), 5.63 (s, 1H, N-C*H*_2_), 5.26 (dd, ^3^*J*_(H5–H4__β__)_ = 6.4 Hz, ^3^*J*_(H5–H4__α__)_ = 6.4 Hz, 1H, *H*C5), 4.34–4.22 (m, 4H, 2 × C*H*_2_OP), 3.41–3.39 (m, 1H, C3), 3.07–3.00 (m, 1H, *H*_β_C4), 3.06 (s, 3H, C*H*_3_-N), 2.98 (dddd, ^3^*J*_(H4__α__–P)_ = 13.4 Hz, ^2^*J*_(H4__α__–H4β__)_ = 13.4 Hz, ^3^*J*_(H4__α__–H3)_ = 10.4 Hz, ^3^*J*_(H4__α__–H5)_ = 6.4 Hz, 1H, *H*_α_C4), 1.42 (t, ^3^*J* = 7.0 Hz, 3H, C*H*_3_CH_2_OP), 1.40 (t, ^3^*J* = 7.1 Hz, 3H, C*H*_3_CH_2_OP). ^13^C-NMR (151 MHz, CDCl_3_): δ = 165.94 (C=O), 163.26, 162.84 (d, ^1^*J*_(CF)_ = 247.6 Hz, C4″), 149.94, 137.25, 131.52 (d, ^4^*J*_(CCCCF)_ = 3.1 Hz, C1″), 130.49 (d, ^3^*J*_(CCCF)_ = 8.0 Hz, C2″, C6″), 129.69, 125.89, 120.75, 116.65, 115.62 (d, ^2^*J*_(CCF)_ = 21.9 Hz, C3″, C5″), 80.09 (d, ^3^*J*_(CCP)_ = 8.1 Hz, C5), 68.40 (N-CH_2_), 64.43 (d, ^1^*J*_(CP)_ = 168.3 Hz, C3), 63.26 (d, ^2^*J*_(COP)_ = 6.5 Hz, CH_2_OP), 62.45 (d, ^2^*J*_(COP)_ = 6.8 Hz, CH_2_OP), 46.59 (CH_3_N), 37.87 (C4), 16.56 (d, ^3^*J*_(CCOP)_ = 5.8 Hz, *C*H_3_CH_2_OP), 16.46 (d, ^3^*J*_(CCOP)_ = 5.3 Hz, *C*H_3_CH_2_OP). ^31^P-NMR (243 MHz, CDCl_3_): δ = 22.00. Anal. Calcd. for C_23_H_26_BrFN_3_O_5_P: C, 49.83; H, 4.73; N, 7.58. Found: C, 50.20; H, 4.62; N, 7.32.

*Diethyl trans-{5-[6-bromo-3-(2,4-difluorobenzyl)-4-oxo-3,4-dihydroquinazolin-2-yl]-2-methylisoxazolidin-3-yl}phosphonate* (*trans*-**11i**). Data presented below were extracted from spectra of a 92:8 mixture of trans-**11i** and cis-**11i**. Yellowish oil. IR (film, cm^−1^) ν_max_: 3079, 2959, 2924, 2853, 1738, 1689, 1613, 1565, 1509, 1490, 1416, 1351, 1280, 1141, 1100, 961, 836, 798. ^1^H-NMR (600 MHz, CDCl_3_): δ = 8.30 (d, ^4^*J* = 2.1 Hz, 1H, HC5′), 7.92 (dd, ^3^*J* = 8.8 Hz, ^4^*J* = 2.1 Hz, 1H, HC7′), 7.85 (d, ^3^*J* = 8.8 Hz, 1H, HC8′), 7.59–7.55 (m, 1H), 6.95–6.89 (m, 2H), 5.68 (AB, *J*_AB_ = 13.3 Hz, 1H, N-CH_2b_), 5.66 (AB, *J*_AB_ = 13.3 Hz, 1H, N-CH_2a_), 5.25 (dd, ^3^*J*_(H5–H4β)_ = 7.7 Hz, ^3^*J*_(H5–H4α)_ = 6.5 Hz, 1H, HC5), 4.33–4.18 (m, 4H, 2 × CH_2_OP), 3.41–3.38 (m, 1H, HC3), 3.07–3.01 (m, 1H, H_β_C4), 3.05 (s, 3H, CH_3_-N), 2.98 (dddd, ^3^*J*_(H4α–P)_ = 12.8 Hz, ^2^*J*_(H4α–H4β)_ = 12.8 Hz, ^3^*J*_(H4α–H3)_ = 9.7 Hz, ^3^*J*_(H4α–H5)_ = 6.5 Hz, 1H, H_α_C4), 1.41 (t, ^3^*J* = 7.0 Hz, 3H, CH_3_CH_2_OP), 1.40 (t, ^3^*J* = 7.0 Hz, 3H, CH_3_CH_2_OP). ^13^C-NMR (151 MHz, CDCl_3_): δ = 165.82 (C=O), 163.32 (dd, ^1^*J*_(CF)_ = 250.9 Hz, ^3^*J*_(CCCF)_ = 12.1 Hz, C2″), 163.20, 161.50 (dd, ^1^*J*_(CF)_ = 251.4 Hz, ^3^*J*_(CCCF)_ = 12.2 Hz, C4″), 149.87, 137.31, 132.09 (dd, ^3^*J*_(CCCF)_ = 9.8 Hz, ^3^*J*_(CCCF)_ = 4.9 Hz, C6″), 129.71, 125.87, 120.81, 118.98 (dd, ^2^*J*_(CCF)_ = 14.4 Hz, ^4^*J*_(CCCCF)_ = 3.4 Hz, C1″), 116.58, 111.51 (dd, ^2^*J*_(CCF)_ = 21.1 Hz, ^4^*J*_(CCCCF)_ = 3.5 Hz, C5″), 104.21 (dd, ^2^*J*_(CCF)_ = 25.3 Hz, ^2^*J*_(CCF)_ = 25.4 Hz, C3″), 80.09 (dd, ^3^*J*_(CCCP)_ = 7.9 Hz, C5), 64.43 (d, ^1^*J*_(CP)_ = 168.3 Hz, C3), 63.26 (d, ^2^*J*_(COP)_ = 6.5 Hz, CH_2_OP), 62.47 (d, ^3^*J*_(CCCF)_ = 2.7 Hz, N-CH_2_), 62.43 (d, ^2^*J*_(COP)_ = 6.3 Hz, CH_2_OP), 46.57 (d, ^3^*J*_(CNCP)_ = 4.0 Hz, CH_3_N), 37.89 (C4), 16.56 (d, ^3^*J*_(CCOP)_ = 5.7 Hz, CH_3_CH_2_OP), 16.50 (d, ^3^*J*_(CCOP)_ = 5.6 Hz, CH_3_CH_2_OP). ^31^P-NMR (243 MHz, CDCl_3_): δ = 21.98. Anal. Calcd. for C_23_H_25_BrF_2_N_3_O_5_P × 0.75 H_2_O: C, 47.15; H, 4.56; N, 7.17. Found: C, 46.84; H, 4.18; N, 6.96 (obtained on a 92:8 mixture of *trans*-**11i** and *cis*-**11i**).

*Diethyl trans-{5-[6-bromo-3-methyl-4-oxo-3,4-dihydroquinazolin-2-yl]-2-methylisoxazolidin-3-yl}phosphonate* (*trans*-**11j**). A yellowish oil. IR (film, cm^−1^) ν_max_: 3521, 3477, 2976, 2912, 2855, 1687, 1606, 1470, 1308, 1265, 1050, 1023, 972, 849, 574. ^1^H-NMR (600 MHz, CDCl_3_): δ = 8.42 (d, ^4^*J* = 2.3 Hz, 1H, *H*C5′), 7.82 (dd, ^3^*J* = 8.7 Hz, ^4^*J* = 2.3 Hz, 1H, *H*C7′), 7.56 (d, ^3^*J* = 8.7 Hz, 1H, *H*C8′), 5.18 (dd, ^3^*J*_(H5–H4β)_ = 7.6 Hz, ^3^*J*_(H5–H4α)_ = 5.7 Hz, 1H, *H*C5), 4.29–4.23 (m, 4H, 2 × C*H*_2_OP), 3.75 (s, 3H, CH_3_), 3.71 (dddd, ^3^*J*_(H4α–P)_ = 12.6 Hz, ^2^*J*_(H4α–H4β)_ = 11.3 Hz, ^3^*J*_(H4α–H3)_ = 9.1 Hz, ^3^*J*_(H4α–H5)_ = 5.7 Hz, 1H, *H*_α_C4), 3.35 (ddd, ^3^*J*_(H3–H4α)_ = 9.1 Hz, ^3^*J*_(H3–H4β)_ = 7.6 Hz, ^2^*J*_(H3-P)_ = 2.8 Hz, 1H, *H*C3), 2.87 (s, 3H, C*H*_3_N), 2.80 (dddd, ^3^*J*_(H4β–P)_ = 15.3 Hz, ^2^*J*_(H4β–H4α)_ = 11.3 Hz, ^3^*J*_(H4β–H3)_ = 7.6 Hz, ^3^*J*_(H4β–H5)_ = 7.6 Hz, 1H, *H*_β_C4), 1.41 (t, ^3^*J* = 7.0 Hz, 3H, C*H*_3_CH_2_OP), 1.40 (t, ^3^*J* = 7.1 Hz, 3H, C*H*_3_CH_2_OP). ^13^C-NMR (151 MHz, CDCl_3_): δ = 161.31 (C=O), 152.70, 145.34, 137.33, 129.44, 129.37, 122.31, 121.02, 76.29 (d, ^3^*J*_(CCCP)_ = 7.8 Hz, C5), 64.35 (d, ^1^*J*_(CP)_ = 170.1 Hz, C3), 62.89 (d, ^2^*J*_(COP)_ = 6.6 Hz, CH_2_OP), 62.73 (d, ^2^*J*_(COP)_ = 7.2 Hz, CH_2_OP), 47.15 (d, ^3^*J*_(CNCP)_ = 6.4 Hz CH_3_N), 34.38 (C4), 30.97 (CH_3_), 16.56 (d, ^3^*J*_(CCOP)_ = 4.9 Hz, *C*H_3_CH_2_OP), 16.53 (d, ^3^*J*_(CCOP)_ = 5.1 Hz, *C*H_3_CH_2_OP). ^31^P-NMR (243 MHz, CDCl_3_): δ = 21.93. Anal. Calcd. for C_17_H_23_BrN_3_O_5_P: C, 44.36; H, 5.04; N, 9.13. Found: C, 44.15; H, 4.75; N, 9.03.

*Diethyl trans-{5-[6-bromo-3-ethyl-4-oxo-3,4-dihydroquinazolin-2-yl]-2-methylisoxazolidin-3-yl}phosphonate* (*trans*-**11k**). Colorless oil. IR (film, cm^−1^) ν_max_: 3055, 2981, 2929, 2854, 1687, 1613, 1569, 1493, 1430, 1383, 1285, 1241, 1117, 1056, 1024, 967, 836. ^1^H-NMR (600 MHz, CDCl_3_): δ = 8.32 (d, ^4^*J* = 2.2 Hz, 1H, *H*C5′), 7.90 (dd, ^3^*J* = 8.8 Hz, ^4^*J* = 2.2.Hz, 1H, *H*C7′), 7.82 (d, ^3^*J* = 8.8 Hz, 1H, *H*C8′), 5.23 (dd, ^3^*J*_(H5–H4β)_ = 8.0 Hz, ^3^*J*_(H5–H4α)_ = 6.0 Hz, 1H, *H*C5), 4.68 (q, ^3^*J* = 7.2 Hz, 2H, CH_3_C*H*_2_), 4.33–4.21 (m, 4H, 2 × C*H*_2_OP), 3.45–3.43 (m, 1H, *H*C3), 3.07 (s, 3H, C*H*_3_N), 3.06–3.01 (m, 1H, *H*_β_C4), 2.97 (dddd, ^3^*J*_(H4α–P)_ = 12.6 Hz, ^2^*J*_(H4α–H4β)_ = 12.6 Hz, ^3^*J*_(H4α–H3)_ = 9.4 Hz, ^3^*J*_(H4α–H5)_ = 6.0 Hz, 1H, *H*_α_C4), 1.55 (t, ^3^*J* = 7.2 Hz, 3H, C*H*_3_CH_2_), 1.42 (t, ^3^*J* = 7.0 Hz, 3H, C*H*_3_CH_2_OP), 1.39 (t, ^3^*J* = 6.8 Hz, 3H, C*H*_3_CH_2_OP). ^13^C-NMR (151 MHz, CDCl_3_): δ =166.33 (C=O), 163.63, 149.78, 137.01, 129.63, 126.03, 120.47, 116.78, 80.22 (d, ^3^*J*_(CCCP)_ = 7.9 Hz, C5), 64.38 (d, ^1^*J*_(CP)_ = 168.0 Hz, C3), 63.28 (d, ^2^*J*_(COP)_ = 6.4 Hz, CH_2_OP), 62.41 (d, ^2^*J*_(COP)_ = 6.7 Hz, CH_2_OP), 47.21 (CH_3_N), 38.01 (C4), 29.69 (CH_3_*C*H_2_) 16.56 (d, ^3^*J*_(CCOP)_ = 5.5 Hz, *C*H_3_CH_2_OP), 16.50 (d, ^3^*J*_(CCOP)_ = 6.2 Hz, *C*H_3_CH_2_OP), 14.29 (*C*H_3_CH_2_). ^31^P-NMR (243 MHz, CDCl_3_): δ = 22.08. Anal. Calcd. for C_18_H_25_BrN_3_O_5_P: C, 45.58; H, 5.31; N, 8.86. Found: C, 45.37; H, 5.31; N, 8.54.

### 3.6. Antiviral Activity Assays

The compounds were evaluated against different herpesviruses, including herpes simplex virus type 1 (HSV-1) strain KOS, thymidine kinase-deficient (TK^−^) HSV-1 KOS strain resistant to ACV (ACV^r^), herpes simplex virus type 2 (HSV-2) strain G, varicella-zoster virus (VZV) strain Oka, TK^−^ VZV strain 07-1, human cytomegalovirus (HCMV) strains AD-169 and Davis, as well as vaccinia virus, adeno virus-2, vesicular stomatitis virus, para-influenza-3 virus, reovirus-1, Sindbis virus, Coxsackie virus B4, Punta Toro virus, respiratory syncytial virus (RSV), feline coronovirus (FIPV), and influenza A virus subtypes H1N1 (A/PR/8), H3N2 (A/HK/7/87) and influenza B virus (B/HK/5/72). The antiviral assays were based on the inhibition of virus-induced cytopathicity or plaque formation in human embryonic lung (HEL) fibroblasts, African green monkey kidney cells (Vero), human epithelial cervix carcinoma cells (HeLa), Crandell-Rees feline kidney cells (CRFK), or Madin Darby canine kidney cells (MDCK). Confluent cell cultures in microtiter 96-well plates were inoculated with 100 CCID_50_ of virus (1 CCID_50_ being the virus dose to infect 50% of the cell cultures) or with 20 plaque forming units (PFU) and the cell cultures were incubated in the presence of varying concentrations of the test compounds. Viral cytopathicity or plaque formation (VZV) was recorded as soon as it reached completion in the control virus-infected cell cultures that were not treated with the test compounds. Antiviral activity was expressed as the EC_50_ or compound concentration required to reduce virus-induced cytopathicity or viral plaque formation by 50%. Cytotoxicity of the test compounds was expressed as the minimum cytotoxic concentration (MCC) or the compound concentration that caused a microscopically detectable alteration of cell morphology. Alternatively, the cytostatic activity of the test compounds was measured based on inhibition of cell growth. HEL cells were seeded at a rate of 5 × 10^3^ cells/well into 96-well microtiter plates and allowed to proliferate for 24 h. Then, medium containing different concentrations of the test compounds was added. After three days of incubation at 37 °C, the cell number was determined with a Coulter counter. The cytostatic concentration was calculated as the CC_50_, or the compound concentration required to reduce cell proliferation by 50% relative to the number of cells in the untreated controls.

### 3.7. Cytostatic Activity against Immortalized Cell Lines

Murine leukemia (L1210), human T-lymphocyte (CEM), human cervix carcinoma (HeLa), and immortalized human dermal microvascular endothelial cells (HMEC-1) were suspended at 300,000–500,000 cells/mL of culture medium, and 100 μL of a cell suspension was added to 100 μL of an appropriate dilution of the test compounds in 200 μL-wells of 96-well microtiter plates. After incubation at 37 °C for two (L1210), three (CEM), or four (HeLa) days, the cell number was determined using a Coulter counter. The IC_50_ was defined as the compound concentration required to inhibit cell proliferation by 50%.

## 4. Conclusions

A series of isoxazolidine-conjugates of quinazolinones *trans*-**11a**–**k** and *cis*-**11a**–**k** were obtained from *N*-methyl *C*-(diethoxyphosphoryl)nitrone and selected N3-substituted 6-bromo-2-vinylquinazolinones. The *trans*-isoxazolidine cycloadducts (*trans*-**11a**, *trans*-**11b**, *trans*-**11c**, *trans*-**11d**, *trans*-**11e**, *trans*-**11g**, *trans*-**11h**, *trans*-**11j**, *trans*-**11k**) and isoxazolidine *cis***-11a**, or the respective mixtures of isoxazolidines (*trans*-**11f/***cis*-**11f** (95:5) and *trans*-**11i/***cis*-**11i** (92:8)) were evaluated for their antiviral activity toward variety of DNA and RNA viruses. Almost all compounds were active against VZV and among them *trans*-**11b**, *trans*-**11d**, *trans*-**11g**, and *trans*-**11h** were the most potent (EC_50_ = 5.8–11.6 μΜ) and, at the same time, exhibited lower cytotoxicity toward uninfected cell lines (CC_50_ = 33–42 μΜ). Furthermore, (isoxazolidine)phosphonates *trans*-**11d**, *trans*-**11g**, *trans*-**11h**, and *trans*-**11i**/*cis*-**11i** (92:8) showed the highest activity against HCMV (EC_50_ = 8.9–12.5 μΜ). On the other hand, several compounds exhibited moderate cytostatic effect toward the CEM cell line (IC_50_ = 9.6–17 μM), however, slightly higher than that of 5-fluorouracil used as the reference drug.

It was proved that installation of functionalized benzyl groups at N3 in the quinazolinone moiety is essential for inhibitory properties toward both VZV and HCMV. At the same time, it was noticed that incorporation of the bromine atom at C6 in a quinazolinone skeleton resulted in a significant increase in potency of isoxazolidine-conjugates **11b**–**11i** toward both VZV (up to five-fold higher) and HCMV (up to three-fold higher) when compared with previously described analogous conjugates **10b**–**10i** lacking a bromine substituent at C6 of the quinazolinone moiety. Moreover, inhibitory properties of the newly synthesized compounds **11b**–**11k** toward tested cell lines were also slightly higher than those of previously described analogues **10b**–**10k**.

## Figures and Tables

**Table 1:** Isoxazolidines *trans*-**11** and *cis*-**11** obtained according to Scheme 3.

Entry	Quinazolinone 13	Ratio of *trans*-11:*cis*-11	Yield (%)
	R		
**a**	H	92:8	*trans*-**11a** (23) ^a^ + *cis*-**11a** (5) ^a^ + *trans*-**11a** and *cis*-**11a** (62) ^b^
**b**	C_6_H_5_-CH_2_	92:8	*trans*-**11b** (8) ^a^ + *trans*-**11b** and *cis*-**11b** (83) ^b^
**c**	2-NO_2_-C_6_H_4_-CH_2_	94:6	*trans*-**11c** (30) ^a^ + *trans*-**11c** and *cis*-**11c** (62) ^b^
**d**	3-NO_2_-C_6_H_4_-CH_2_	92:8	*trans*-**11d** (14) ^a^ + *trans*-**11d** and *cis*-**11d** (80) ^b^
**e**	4-NO_2_-C_6_H_4_-CH_2_	90:10	*trans*-**11e** (18) ^a^ + *trans*-**11e** and *cis*-**11e** (72) ^b^
**f**	2-F-C_6_H_4_-CH_2_	90:10	*trans*-**11f** and *cis*-**11f** (96) ^b^
**g**	3-F-C_6_H_4_-CH_2_	92:8	*trans*-**11g** (10) ^a^ + *trans*-**11g** and *cis*-**11g** (72) ^b^
**h**	4-F-C_6_H_4_-CH_2_	92:8	*trans*-**11h** (12) ^a^ + *trans*-**11h** and *cis*-**11h** (86) ^b^
**i**	2,4-diF-C_6_H_3_-CH_2_	93:7	*trans*-**11i** and *cis*-**11i** (82) ^b^
**j**	CH_3_	94:6	*trans*-**11j** (32) ^a^ + *trans*-**11j** and *cis*-**11j** (65) ^b^
**k**	CH_3_CH_2_	88:12	*trans*-**11k** (14) ^a^ + *trans*-**11k** and *cis*-**11k** (80) ^b^

^a^ yield of pure isomer, ^b^ yield of pure mixture of *cis*- and *trans*-isomers.

**Table 2:** Antiviral activity and cytotoxicity against varicella-zoster virus (VZV) in HEL cell cultures.

Compound	R	Antiviral Activity EC_50_ (μM) ^a^		Cytotoxicity (μM)	
		TK^+^ VZV Strain (OKA)	TK^−^ VZV Strain (07-1)	Cell Morphology MCC ^b^	Cell Growth CC_50_^c^
*cis*-**11a**	H	>100	>100	>100	n.d.
*trans*-**11a**	H	>100	66.87	>100	n.d.
*trans*-**11b**	C_6_H_5_-CH_2_	13.5 ± 7.1	13.6 ± 9.1	100	42.3 ± 0.3
*trans*-**11c**	2-NO_2_-C_6_H_4_-CH_2_	10.3 ± 1.1	5.4 ± 1.0	100	9.4 ± 0.4
*trans*-**11d**	3-NO_2_-C_6_H_4_-CH_2_	8.3 ± 1.4	5.8 ± 1.4	100	37.0 ± 4.3
*trans*-**11e**	4-NO_2_-C_6_H_4_-CH_2_	6.84	7.51	20	n.d.
*trans*-**11f**/*cis*-**11f** (95:5)	2-F-C_6_H_4_-CH_2_	7.76	9.56	20	n.d.
*trans*-**11g**	3-F-C_6_H_4_-CH_2_	11.6 ± 5.3	7.7 ± 6.2	>20	33.0 ± 3.5
*trans*-**11h**	4-F-C_6_H_4_-CH_2_	12.6 ± 2.6	7.5 ± 5.4	>20	39.4 ± 5.2
*trans*-**11i**/*cis*-**11i** (92:8)	2,4-diF-C_6_H_3_-CH_2_	8.7 ± 3.2	10.5 ± 0.3	>20	15.6 ± 3.2
*trans*-**11j**	CH_3_	>4	>4	20	n.d.
*trans*-**11k**	CH_3_CH_2_	>20	>20	100	n.d.
**Acyclovir**		1.55 ± 1.0	39.2 ± 3.6	>440	>350
**Brivudin**		0.023 ± 0.008	31.9 ± 16.1	>300	>300

^a^ Effective concentration required to reduce virus plaque formation by 50%. Virus input was 100 plaque forming units (PFU), ^b^ Minimum cytotoxic concentration that causes a microscopically detectable alternation of cell morphology, ^c^ Cytotoxic concentrations required to reduce cell growth by 50%, n.d.—not determined.

**Table 3:** Antiviral activity and cytotoxicity against human cytomegalovirus in HEL cell cultures.

Compound	R	Antiviral Activity EC_50_ (μM) ^a^		Cytotoxicity (μM)	
		AD-169 Strain	Davis Strain	Cell Morphology MCC ^b^	Cell Growth CC_50_^c^
*cis*-**11a**	H	>100	100	20	n.d.
*trans*-**11a**	H	>100	>100	100	n.d.
*trans*-**11b**	C_6_H_5_-CH_2_	20	20	20	n.d.
*trans*-**11c**	2-NO_2_-C_6_H_4_-CH_2_	>20	15.29	100	n.d.
*trans*-**11d**	3-NO_2_-C_6_H_4_-CH_2_	10.4 ± 0.8	11.6 ± 2.5	100	37.0 ± 4.3
*trans*-**11e**	4-NO_2_-C_6_H_4_-CH_2_	>20	>20	100	n.d.
*trans*-**11f**/*cis*-**11f** (95:5)	2-F-C_6_H_4_-CH_2_	8.94	8.94	20	n.d.
*trans*-**11g**	3-F-C_6_H_4_-CH_2_	10.5 ± 2.2	8.94 ± 0	100	33.0 ± 3.5
*trans*-**11h**	4-F-C_6_H_4_-CH_2_	12.5 ± 3.9	11.2 ± 3.1	100	39.4 ± 5.2
*trans*-**11i**/*cis*-**11i** (92:8)	2,4-diF-C_6_H_3_-CH_2_	9.4 ± 0.46	9.7 ± 1.1	>20	15.6 ± 3.2
*trans*-**11j**	CH_3_	>20	>100	20	n.d.
*trans*-**11k**	CH_3_CH_2_	>20	44.72	20	n.d.
**Ganciclovir**		16.9 ± 6.9	7.7 ± 0.9	>350	>350
**Cidofovir**		1.5 ± 0.2	1.7 ± 0.4	>300	>300

^a^ Effective concentration required to reduce virus plaque formation by 50%. Virus input was 100 plaque forming units (PFU), ^b^ Minimum cytotoxic concentration that causes a microscopically detectable alternation of cell morphology, ^c^ Cytotoxic concentrations required to reduce cell growth by 50%, n.d.—not determined.

**Table 4:** Inhibitory effect of the tested compounds against the proliferation of murine leukemia (L1210), human T-lymphocyte (CEM), human cervix carcinoma (HeLa), and immortalized human dermal microvascular endothelial cells (HMEC-1).

Compound	R	IC_50_^a^ (μM)			
		L1210	CEM	HeLa	HMEC-1
*cis*-**11a**	H	>250	242 ± 7	>250	140 ± 54
*trans*-**11a**	H	126 ± 19	148 ± 2	196 ± 20	85 ± 13
*trans*-**11b**	C_6_H_5_-CH_2_	20 ± 2	16 ± 3	26 ± 7	30 ± 0
*trans*-**11c**	2-NO_2_-C_6_H_4_-CH_2_	19 ± 3	14 ± 4	22 ± 11	30 ± 0
*trans*-**11d**	3-NO_2_-C_6_H_4_-CH_2_	26 ± 1	33 ± 25	24 ± 9	30 ± 0
*trans*-**11e**	4-NO_2_-C_6_H_4_-CH_2_	18 ± 76	17 ± 12	24 ± 9	26 ± 6
*trans*-**11f**/*cis*-**11f** (95:5)	2-F-C_6_H_4_-CH_2_	17 ± 2	13 ± 1	25 ± 7	30 ± 0
*trans*-**11g**	3-F-C_6_H_4_-CH_2_	16 ± 2	10 ± 0	25 ± 7	30 ± 0
*trans*-**11h**	4-F-C_6_H_4_-CH_2_	16 ± 0	9.6 ± 2.2	25 ± 7	29 ± 1
*trans*-**11i**/*cis*-**11i** (92:8)	2,4-diF-C_6_H_3_-CH_2_	16 ± 1	9.8 ± 4.8	22 ± 6	30 ± 0
*trans*-**11j**	CH_3_	138 ± 19	128 ± 21	175 ± 105	>250
*trans*-**11k**/*cis*-**11k** (97:3)	CH_3_CH_2_	102 ± 14	44 ± 18	113 ± 44	121 ± 41
**5-Fluorouracil**		0.33 ± 0.17	18 ± 5	0.54 ± 0.12	n.d.

^a^ 50% Inhibitory concentration or compound concentration required to inhibit tumor cell proliferation by 50%, n.d.—not determined.
